# The burden of headache disorders in the Eastern Mediterranean Region, 1990-2016: findings from the Global Burden of Disease study 2016

**DOI:** 10.1186/s10194-019-0990-3

**Published:** 2019-04-25

**Authors:** Kia Vosoughi, Lars Jacob Stovner, Timothy J. Steiner, Maziar Moradi-Lakeh, Seyed-Mohammad Fereshtehnejad, Farshad Farzadfar, Pouria Heydarpour, Reza Malekzadeh, Mohsen Naghavi, Mohammad Ali Sahraian, Sadaf G. Sepanlou, Arash Tehrani-Banihashemi, Reza Majdzadeh, Valery L. Feigin, Theo Vos, Ali H. Mokdad, Christopher J. L. Murray

**Affiliations:** 1 Preventive Medicine and Public Health Research Center, Iran University of Medical Sciences, Tehran, Iran; 2 Department of Neuromedicine and Movement Science (INB), Norwegian University of Science and Technology (NTNU), Trondheim, Norway; 3 Department of Neurology and Clinical Neurophysiology, Norwegian Advisory Unit on Headache, St Olavs University Hospital, Trondheim, Norway; 4 Division of Brain Sciences, Imperial College London, London, UK; 5 Lifting The Burden, London, UK; 6 Division of Neurology, Department of Medicine, The Ottawa Hospital, University of Ottawa, Ottawa, ON Canada; 7 Division of Clinical Geriatrics, Department of Neurobiology, Care Sciences and Society (NVS), Karolinska Institutet, Stockholm, Sweden; 8 Non-Communicable Diseases Research Center, Endocrinology and Metabolism Population Sciences Institute, Tehran University of Medical Sciences, Tehran, Iran; 9 Multiple Sclerosis Research Center, Tehran University of Medical Sciences, Tehran, Iran; 10 Digestive Disease Research Institute, Tehran University of Medical Sciences, Tehran, Iran; 11 Department of Health Metrics Sciences, University of Washington, Seattle, WA USA; 12 Preventive Medicine and Public Health Research Center, Social Health Institute, Iran University of Medical Sciences, Tehran, Iran; 13 Department of Community Medicine, School of Medicine, Iran University of Medical Sciences, Tehran, Iran; 14 Knowledge Utilization Research Center, Tehran University of Medical Sciences, Tehran, Iran; 15 National Institute for Stroke and Applied Neurosciences, Auckland University of Technology, Auckland, New Zealand; 16 Department of Medicine, Johns Hopkins Medical Institutions, Baltimore, MD USA

**Keywords:** Primary headache, Migraine, Tension-type headache, Burden, Prevalence, Years lived with disability (YLDs), Disability-adjusted life years (DALYs), Global burden of disease study (GBD), Eastern Mediterranean region (EMR)

## Abstract

**Objectives:**

Using the findings of the Global Burden of Disease Study (GBD), we report the burden of primary headache disorders in the Eastern Mediterranean Region (EMR) from 1990 to 2016.

**Methods:**

We modelled headache disorders using DisMod-MR 2.1 Bayesian meta-regression tool to ensure consistency between prevalence, incidence, and remission. Years lived with disability (YLDs) were calculated by multiplying prevalence and disability weight (DW) of migraine and tension-type headache (TTH). We assumed primary headache disorders as non-fatal, so their YLD is equal to disability-adjusted life years (DALYs).

**Results:**

Migraine and TTH were the second and twentieth leading causes of YLDs in EMR. Between 1990 and 2016, the absolute YLD numbers of migraine and TTH increased from 2.3 million (95% uncertainty interval (UI): 1.5–3.2) to 4.7 million (95%UI: 3–6.5) and from 383 thousand (95%UI: 240–562) to 816 thousand (95%UI: 516–1221), respectively. During the same period, age-standardised YLD rates of migraine and TTH in EMR increased by 0.7% and 2.5%, respectively, in comparison to a small decrease in the global rates (0.2% decrease in migraine and TTH). The bulk of burden due to headache occurred in the 30–49 year age group, with a peak at ages 35–44 years. The age-standardised YLD rates of both headache disorders were higher in women with female to male ratio of 1.69 for migraine and 1.38 for TTH. All countries of the EMR except for Somalia and Djibouti had higher age-standardised YLD rates for migraine and TTH in compare to the global rates. Libya and Saudi Arabia had the highest increase in age-standardised YLD rates of migraine and TTH, respectively.

**Conclusion:**

The findings of this study show that primary headache disorders are a major and a growing cause of disability in EMR. Since 1990, burden of primary headache disorders has constantly been higher in EMR compared to rest of the world, which indicates that health systems in EMR must focus further on developing and implementing preventive and management strategies to control headache.

**Electronic supplementary material:**

The online version of this article (10.1186/s10194-019-0990-3) contains supplementary material, which is available to authorized users.

## Introduction

Headache, with the global prevalence of 47%, is one of the most common neurological disorders [1] Headache can be a secondary symptom of an underlying conditions (e.g. intracranial neoplasms, epileptic seizures, and intracranial infections); however, in most cases it is a primary and non-fatal disorder [2, 3]. Primary headaches such as migraine, tension-type headache (TTH), and cluster headache, although non-fatal, cause substantial disability and economic cost [4–7].

Global Burden of Diseases, Injuries and Risk Factors study (GBD) is a comprehensive worldwide epidemiologic study being performed since 1990 [8, 9]. Estimation of years lived with disability (YLDs) and prevalence of diseases were among the main objectives of GBD [4]. In GBD 2000, for the first time, migraine was included as the only headache subtype. Since then, TTH was added to the GBD study, and some of the limitations in the previous estimates were addressed by using more comprehensive and reliable epidemiologic data based on population-based surveys from countries without previous available data [4]. GBD 2016, provided more accurate estimations of prevalence and burden of headache by countries, regions, and super regions [4].

According to GBD 2016, prevalence of headache disorders was variable across different geographic regions. For example, migraine was less frequent in African and Western Pacific WHO regions and TTH was less frequent in African region. However, the overall global all-age prevalence of migraine and TTH were estimated to be 14.1% (95% uncertainty interval [UI] 13.5–14.8%), and 25.6% (95% UI 23.1–28.4) respectively in 2016. Although prevalence of headache is an important epidemiologic measure, the burden of disability related to headache, as measured by YLD, is more informative for health policy making. Globally, migraine, with 45,121,909 YLDs (95% UI 29,045,835-62,826,904), and TTH, with 7,195,122 YLDs (95% UI 4,614,628-10,499,903), were the second and 28 leading causes of disability.

Despite the considerable burden, headache disorders are still underestimated and underdiagnosed [1, 10]. A study has estimated that the headache diagnosis rate is under 40% [11], which leads to a high undertreated rate. Effective treatment for most primary headache cases can be provided by primary health service and with low cost [11]; therefore, improvement of the diagnosis and treatment rate will substantially reduce the burden of headache.

GBD 2016 emphasized that primary headache disorders are an important health priority. Estimating the burden of headache is the first step to implement further measures to reduce its burden such as educating health care providers, developing primary care management, and allocating resources. In this study, we reported the prevalence and burden of primary headache disorders (including migraine and TTH) in Eastern Mediterranean Region (EMR) countries from 1990 to 2016 using data and methods of the Global Burden of Diseases, Injuries, and Risk Factors Study 2016.

## Methods

### Overview

The Global Burden of Diseases, Injuries, and Risk Factors Study 2016 (GBD 2016) is a standardised analytical method that used all eligible sources to estimate the epidemiological data, including prevalence, mortality, years of life lost (YLL), YLDs, and disability-adjusted life years (DALYs), for 328 causes by sex, age, and location from 1990 to 2016. It estimates all parameters for 195 countries and territories, nested in twenty-one regions and seven super-regions. Details of the methodology of GBD studies and the main changes applied in GBD 2016 has been explained in another article [4].

### Locations, causes, and parameters

The World Health Organization (WHO) EMR, home to approximately 600 million people [12], contains 22 countries: Afghanistan, Bahrain, Djibouti, Egypt, Iran, Iraq, Jordan, Kuwait, Lebanon, Libya, Morocco, Oman, Pakistan, Palestine, Qatar, Saudi Arabia, Somalia, Sudan, Syria, Tunisia, United Arab Emirates, and Yemen. In this article all epidemiologic parameters have been reported for the whole EMR region and all countries separately.

From the category of neurological disorders, we included migraine and tension-type headache (TTH) - the two types of primary headache disorders which were covered in GBD 2016. In the previous GBD iteration (GBD 2015), in addition to migraine and TTH, medication overuse headache (MOH) was also included as a separate disorder. In the present iteration, MOH has been removed as an independent cause, and the YLDs of MOH has been calculated as a sequel of migraine and TTH. Based on the previous studies, 73 % of total MOH YLDs were classified as a sequel of migraine (MOH due to migraine) and the rest of them were classified as a sequel of TTH (MOH due to TTH) [13–15]. Secondary headache disorders were not included in this study.

In this article, we presented numbers and rates of prevalence and YLDs of migraine and TTH in 2016 and the changes from 1990 to 2016 for all EMR countries. We assumed that primary headache disorders do not lead to mortality and, therefore, disability adjusted life-years (DALYs) of migraine and TTH are equal to their YLDs.

### Data sources

In GBD 2016, systematic review for migraine, TTH, and MOH were updated as part of the GBD standard methodology. Details on the data sources have been described elsewhere [16]. From the EMR, data sources from Iran [17–19], Pakistan [20], Tunisia [21], and UAE [22] for migraine, and data sources from Iran [17, 19, 23], Pakistan [20], and Qatar [24] for TTH were used in GBD 2016; however, data inputs from all over the world were used to model the burden of migraine and TTH in EMR countries.

### Modeling

In order to make data more consistent and suitable for modelling, age-sex splitting was applied to the sources that had reported data by age or sex but not by both age and sex.

Non-fatal modelling, using DisMod-MR 2.1, were performed to estimate prevalence and incidence of migraine, TTH, and MOH. DisMod-MR 2.1 is a Bayesian meta-regression method that estimates non-fatal outcomes using sparse and heterogeneous epidemiological data. It also pools data from different sources and adjusts them for variations in study methods across sources and enforces consistency between different epidemiological parameters. Binary study-level covariates were used in modelling to minimize residual errors of the estimated prevalence and YLDs. Using the mixed-effects nonlinear regression on all the available data at the global level, super-region Bayesian priors were generated; likewise, the super-region regression model was then used to generate region Bayesian priors, and so on down the cascade. Bayesian priors of the EMR countries were generated by using three different super-region models: Eastern Sub-Saharan Africa model for Djibouti and Somalia, South Asia model for Pakistan, and North Africa and Middle East model for the rest of EMR countries. For GBD 2016, the same disability weights (DW) as in GBD 2015 were used. Table 1 displays ICD 10 and ICD 9 codes, sequelae, description, and DW of primary headache disorders.

**Table 1:** ICD 10 codes, ICD 9 codes, sequelae, description, and disability weight of migraine and TTH

Cause	ICD10	ICD9	Sequel	Health state lay description	Disability weight (UI)
Migraine	G43-G43.919	346-346.93			
			Asymptomatic medication overuse headache due to migraine	-	-
			Asymptomatic migraine	-	-
			Symptomatic medication overuse headache due to migraine	Has daily headaches, felt as dull pain and often lasting all day, with poor sleep, nausea and fatigue. The person takes medicine for the headaches, which provides little relief but is needed to avoid having worse symptoms.	0.223 (0.146–0.313)
			Symptomatic migraine	Has severe, throbbing head pain and nausea that cause great difficulty in daily activities and sometimes confine the person to bed. Moving around, light, and noise make it worse.	0.441 (0.294–0.588)
Tension-type headache	G44-G44.89	307.81, 339-339.89			
			Asymptomatic medication overuse headache due to tension-type headache	-	-
			Asymptomatic tension-type headache	-	-
			Symptomatic medication overuse headache due to tension-type headache	Has daily headaches, felt as dull pain and often lasting all day, with poor sleep, nausea and fatigue. The person takes medicine for the headaches, which provides little relief but is needed to avoid having worse symptoms.	0.223 (0.146–0.313)
			Symptomatic tension-type headache	Has a moderate headache that also affects the neck, which causes difficulty in daily activities.	0.037 (0.022–0.057)

*ICD-10* International Statistical Classification of Diseases and Related Health Problems, tenth revision, *ICD-9* International Statistical Classification of Diseases and Related Health Problems, ninth revision

### YLD computation

YLDs were calculated by multiplying prevalence and DW for each sequel, and then YLDs were adjusted for occurrence of simultaneous comorbidities. Comorbidity with additional disorders in a patient with primary headache was estimated by calculating the independent probability of having simultaneous sequelae.

### Uncertainty interval and age-standardised values

We repeated calculation of comorbidity-adjusted YLDs 1000 times and generated a distribution with the 1000 samples. The 25th and 975th values of the 1000 draws determined the upper and lower bounds of the 95% UI. Age-standardised prevalence rate and age-standardised YLD rates were calculated using the GBD reference population [25].

### Socio-demographic Index (SDI) and expected YLD rates on the basis of SDI

SDI was used to provide a comparable metric for overall sociodemographic development. SDI, expressed on a scale of 0 to 1, is a summary measure that identifies where GBD locations sit on the spectrum of socioeconomic development [26]. SDI was calculated based on the geometric mean of lag-distributed income (LDI), average years of schooling among populations aged 15 years or older, and total fertility rate (TFR). Five SDI quintiles, high, high-middle, middle, low-middle, and low, were selected based on SDI values. More details regarding the calculation of SDI are provided in previous GBD publications [4, 27].

Expected YLD rates at each level SDI were generated by a Gaussian regression model [4, 28]. Expected YLD rates on the basis of SDI was compared to the observed values to investigate the performance of countries on the basis of what would be expected on the basis of their overall development.

## Results

### Prevalence

**Fig. 1:**
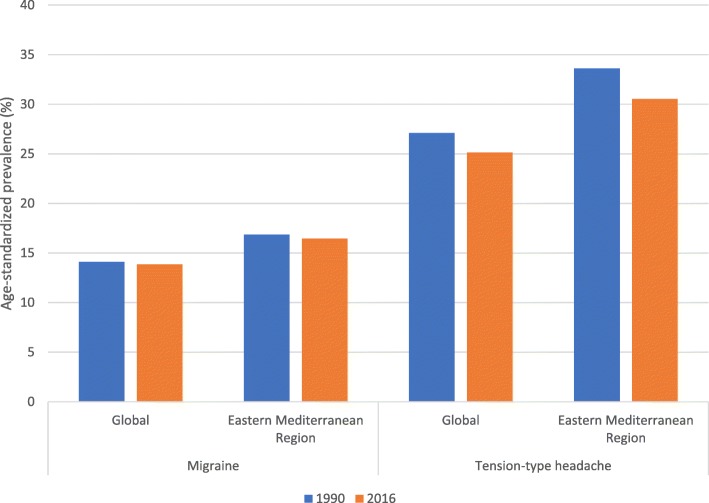
Change in age-standardised prevalence rates from 1990 to 2016. Abbreviations: TTH, tension-type headache; EMR, Eastern Mediterranean Region

Estimated all-age prevalence of TTH and migraine in EMR for 2016 were 29.7% (95% UI 26.8–33) and 15.9% (95% UI 15.2–16.8), respectively. Between 1990 and 2016, the number of individuals with migraine in EMR increased from 53 million (95% UI 50–55 million) to 105 million (95% UI 100–110 million), and the number of individuals with TTH increased from 107 million (95% UI 96–119 million) to 195 million (95% UI 176–217 million). From 1990 to 2016, age-standardised prevalence of migraine and TTH remained generally unchanged both in the EMR and globally, with a constant higher rate in the EMR (Fig. 1).

In the EMR, prevalence of headache was higher in females, with prevalence count female to male ratio of 1.7 and 1.2 for migraine and TTH, respectively. Headache was most common in young and middle-aged adults, with highest prevalence count in 35 to 39 year age group for both migraine (25.9, 95% UI 23.7–28.5%) and TTH (46, 95% UI 33.9–57.8%).

Djibouti had the lowest and Pakistan had the highest prevalence numbers for both migraine and TTH. From 1990 to 2016, UAE had the highest increase in the number of individuals with migraine and TTH (Table 2). In 2016, Pakistan had the highest age-standardised prevalence rate of migraine and Afghanistan had the highest age-standardised prevalence rate of TTH, while Djibouti had the lowest age-standardised prevalence for both migraine and TTH (Table 2). In Web Additional file 1: Table S1 we reported the details of prevalence of headache disorders in EMR countries for sex and age groups from 1990 to 2016.

**Table 2:** Prevalence numbers and rates for 2016, and percentage change in prevalence numbers between 1990 and 2016 for migraine and TTH in EMR countries

Country	Migraine			TTH			SDI
	All-age counts in 2016	Age standardised Rate in 100,000 in 2016	Percentage change in counts between 1990 and 2016	All-age counts in 2016	Age standardised Rate in 100,000 in 2016	Percentage change in counts between 1990 and 2016	
Low-income countries							
Somalia	989744 (924459-1051398)	10984 (10341-11597)	69.7 (64.7-75.8)	2277745 (2009536-2584784)	25025 (22285-27957)	59.5 (54.1-65)	0.27
Afghanistan	4841208 (4533619-5141654)	16769 (15763-17791)	192 (182.2-202.2)	9939058 (8934823-11103449)	34213 (30999-37711)	182.4 (174.1-191.7)	0.28
Djibouti	94816 (88900-100544)	10768 (10140-11410)	73.6 (67.8-79.5)	207419 (183060-234817)	23528 (20954-26280)	55.1 (48.8-62.1)	0.42
Yemen	4113092 (3847322-4385146)	16742 (15743-17824)	164.4 (156.5-172)	8070505 (7225304-9038084)	32528 (29477-36127)	145.6 (136-155.7)	0.45
Middle-income countries							
Palestine	714895 (666691-761373)	16702 (15676-17723)	155.1 (147.5-163.5)	1399904 (1254072-1570053)	32495 (29398-36006)	149.6 (141.4-158.3)	0.45
Iraq	5534181 (5162542-5886763)	16732 (15705-17746)	130.7 (123.5-137.6)	10732501 (9576752-11948005)	32295 (29042-35696)	123 (115.3-131)	0.47
Sudan	6119599 (5731877-6502575)	16681 (15652-17738)	117 (110.3-123.3)	11733161 (10534241-13096213)	31818 (28800-35254)	103 (96.9-109.5)	0.52
Pakistan	30772692 (29418094-32056038)	17159 (16417-17864)	93.6 (89.3-97.5)	56439842 (51246017-62527542)	30802 (28063-34000)	76.2 (69.2-82.7)	0.52
Morocco	5883012 (5509597-6260127)	16641 (15631-17691)	56.5 (51.3-61.9)	10851241 (9726403-12093488)	30786 (27659-34234)	44.2 (38.1-50.7)	0.62
Syria	2864674 (2678877-3057047)	16498 (15470-17620)	68.3 (63.1-73.7)	5310861 (4745133-5968312)	30493 (27353-33932)	54.2 (48.5-60.1)	0.64
Egypt	14623084 (13720844-15493197)	16445 (15461-17417)	78 (72.5-83.6)	26934828 (24165685-30075283)	30282 (27288-33558)	67.6 (61.3-73.2)	0.65
Jordan	1203358 (1124971-1284958)	16300 (15292-17342)	168.9 (159.7-178.1)	2196597 (1951993-2462165)	29747 (26728-33050)	147.7 (136.6-160.1)	0.69
Tunisia	1898520 (1796756-2006354)	15909 (15061-16789)	57 (52.6-61.6)	3545921 (3184205-3944544)	29888 (26900-33130)	44.3 (37.6-51)	0.70
Iran	13616955 (12927168-14325355)	15671 (14948-16443)	82.5 (77.3-87.8)	26496199 (23627819-29527389)	30592 (27572-33761)	61.2 (51-71.6)	0.77
Libya	1054747 (988490-1121886)	16310 (15336-17311)	68.8 (62.6-74.5)	1837414 (1632454-2055257)	28642 (25687-31936)	54.3 (47-61.9)	0.81
Lebanon	1017537 (954285-1084062)	16244 (15220-17299)	139.9 (132.7-146.9)	1795208 (1607600-2009884)	28617 (25722-31958)	128.3 (120.1-136.4)	0.81
High-income countries							
Oman	773924 (720555-827174)	14671 (13764-15561)	210.4 (199.4-221.3)	1476286 (1287874-1688025)	27845 (24857-31048)	178.2 (158.8-197.6)	0.72
Bahrain	240046 (223355-256642)	15150 (14217-16094)	208 (198.9-217.5)	443334 (390844-499432)	28153 (25333-31417)	193.6 (181.6-206.9)	0.74
United Arab Emirates	1566357 (1464258-1672209)	13535 (12800-14302)	484.5 (467.3-501.2)	3050507 (2687218-3465766)	26615 (23875-29831)	446.8 (422.7-474.8)	0.77
Qatar	395766 (367481-425083)	14080 (13198-14994)	430.4 (414.3-445.9)	702549 (610417-805880)	24949 (22401-28113)	421.2 (397-447)	0.79
Saudi Arabia	5797325 (5544243-6045186)	17145 (16433-17856)	132 (128.6-135.7)	8683665 (7709945-9724810)	26066 (23368-29054)	96.6 (88.2-105.3)	0.80
Kuwait	712246 (664472-759904)	15598 (14641-16564)	118 (111.1-125.1)	1250436 (1101203-1414499)	27658 (24743-30881)	106 (96-117.3)	0.83
EMR and global							
EMR	104827778 (99615391-110043848)	16443 (15648-17252)	99.3^a^	195375180 (175945826-216959618)	30520 (27700-33730)	83.0^a^	N/A
Global	1044771478 (999534692-1087968951)	13847 (13255-14418)	48.0^a^	1890670389 (1707786493-2097761629)	25130 (22741-27895)	37.0^a^	0.70

*TTH* tension-type headache, *EMR* eastern Mediterranean region

^a^ Uncertainty interval is not available

### Disability

In 2016, there were an estimated 4.7 million (95% UI 3–6.6 million) YLDs for migraine and 0.8 million (95% UI 0.5–1.2) YLDs for TTH in EMR. We ranked the leading causes of YLDs in 1990 and 2016 at level 4 of the GBD cause hierarchy in EMR. Among the causes with the highest YLDs, migraine had the second position in both 1990 and 2016, and TTH rose from the 25th to the 20th position. During the same period, the relative contribution of migraine YLDs and TTH YLDs to the overall YLDs in EMR increased from 6.0% (95% UI 4.3–7.8) to 6.7% (95% UI 4.9–8.5) and from 1.00% (95% UI 0.75–1.30) to 1.16% (95% UI 0.86–1.49), respectively.

**Fig. 2:**
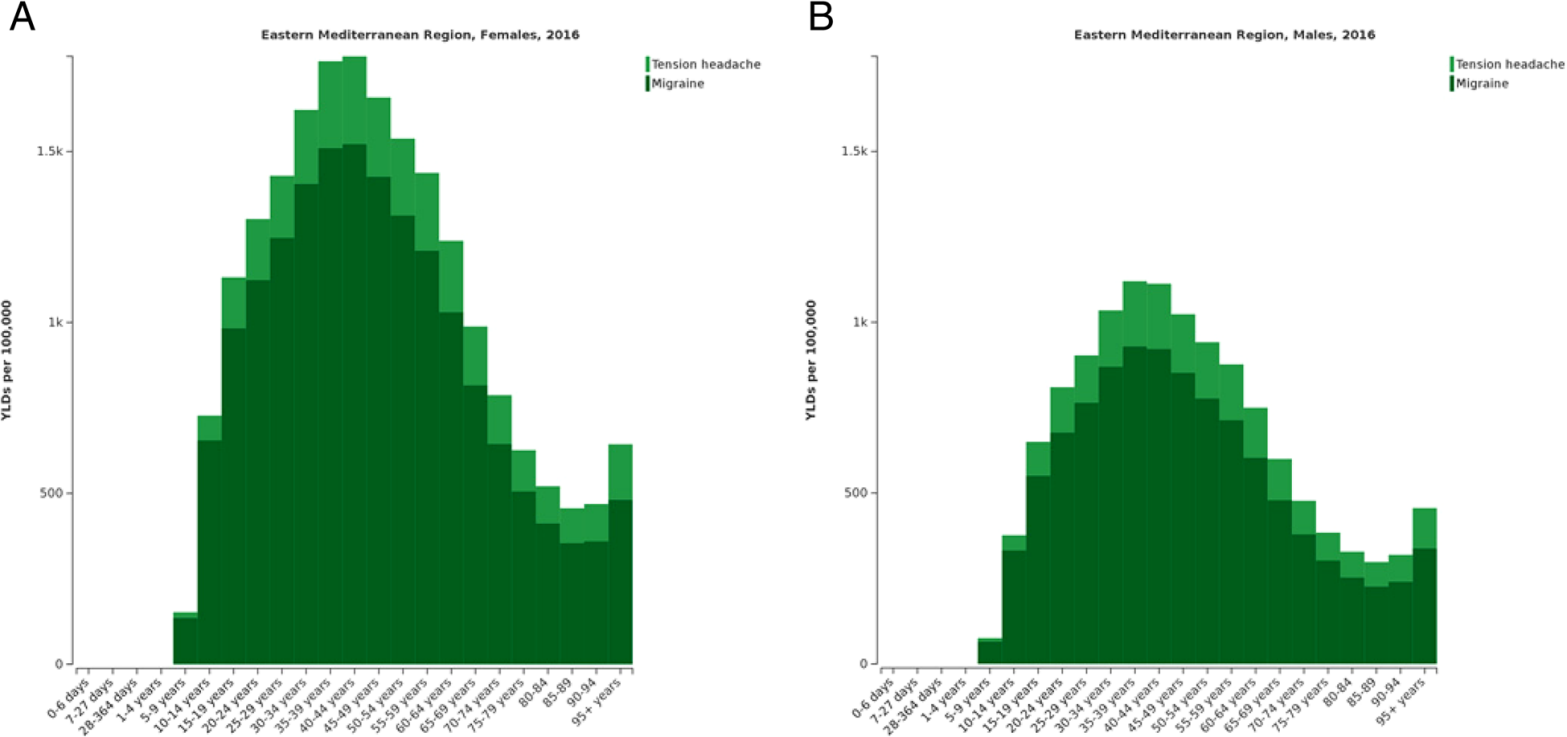
YLD rates of migraine and TTH by age in EMR for females (**a**) and males (**b**) in 2016. Abbreviations: TTH, tension-type headache; EMR, Eastern Mediterranean Region

YLDs from primary headache disorders were highest in young and middle-aged adults. The highest proportion of YLDs due to migraine occurred between ages 30 and 49, with a peak in the 35–44 year age group. The highest proportion of YLDs due to TTH occurred in 30–59 year age group, with a peak in the 35–44 year age group. Across all age groups, YLD rates of both migraine and TTH were considerably higher in females compared to males (Fig. 2). In the EMR, female to male ratio of age-standardised YLDs were 1.69 for migraine and 1.38 for TTH.

**Fig. 3:**
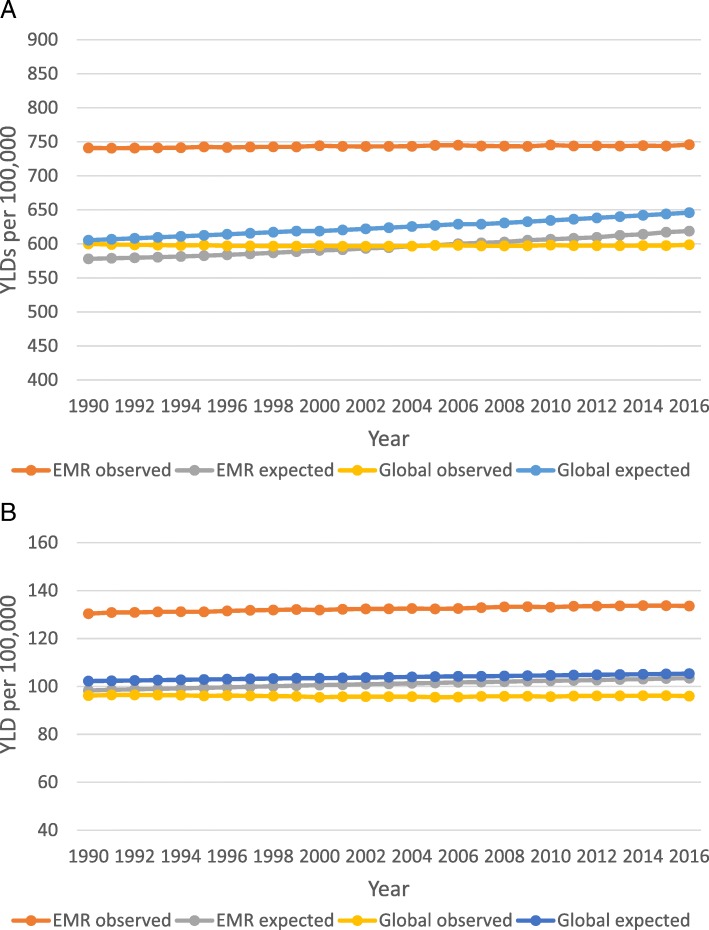
Trends of observed age-standardised YLD rates and expected age-standardised YLD rates on the basis of SDI for migraine (**a**) and TTH (**b**) in the EMR and globally. Abbreviations: TTH, tension-type headache; EMR, Eastern Mediterranean Region

From 1990 to 2016, age-standardised YLD rates of migraine and TTH remained generally unchanged. During this period, EMR had a constantly higher YLD rates compared to the global rates. Since 1990, EMR had greater than expected age-standardised YLD rates on the basis of SDI (observed to expected YLD rates of greater than 1) (Fig. 3).

**Fig. 4:**
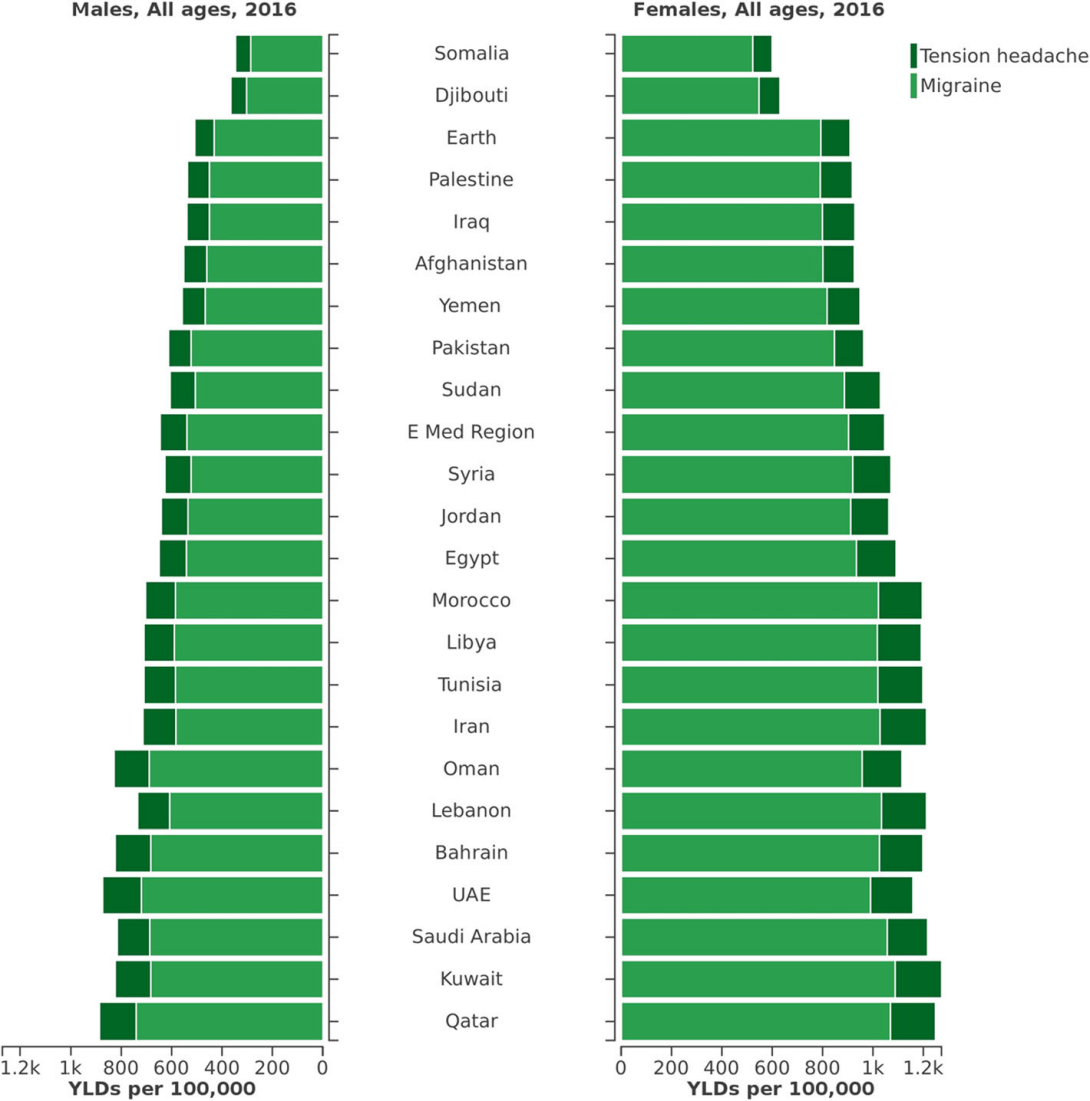
All-age YLD rates for migraine and TTH in the Eastern Mediterranean countries, by country and sex. Abbreviations: YLD, years lived with disability; TTH, tension-type headache; UAE, United Arab Emirate

Comparing the overall all-age YLD rates of migraine and TTH combined, Kuwait had the highest and Djibouti had the lowest YLD rates. Across all EMR countries, all age YLD rates for both migraine and TTH were greater in females rather than males (Fig. 4). In Web Additional file 2: Table S2 we displayed the details of burden of headache disorders in EMR countries for sex and age groups from 1990 to 2016.

For migraine, YLD numbers and rates in 2016, YLD changes between 1990 and 2016, and the ratio of observed to expected YLDs on the basis of SDI in 2016 across EMR countries have been showed in Table 3. Iran, Oman, and Saudi Arabia had the largest, and Somalia had the smallest increase in all-age YLD rates for migraine between 1990 and 2016. During the same period, age-standardised YLD rates of migraine remained generally unchanged in EMR countries. The ratio of observed to expected age-standardised YLD rate for migraine ranged from 0.82 in Djibouti to 1.31 in Palestine. Only three countries -Djibouti, Somalia, and UAE- had lower observed than expected age-standardised YLD rate for migraine, two of them within Eastern Sub-Saharan Africa (SSA). The five locations with the highest age-standardised YLD rates relative to the rates expected on the basis of SDI were Palestine, Iraq, Yemen, Sudan, and Afghanistan. High-income countries in EMR generally had ratios of observed to expected YLD rates close to one.

**Table 3:** YLD count, All-age YLD rates, and Age-standardised rates in 2016, and percentage change of YLD count, All-age YLD rates, and Age-standardised rates between 1990 and 2016 for migraine in EMR countries

Country	All-age counts		Age-standardised rates (per 100,000)				SDI
	2016	Percentage change between 1990 and 2016	2016	Percentage change between 1990 and 2016	Observed to expected ratio	F/M ratio	
Low-income Countries							
Somalia	42078 (27076-59527)	71.1 (63.6 to 78.5)	477 (307-666)	0 (-3.8 to 4)	0.82	1.79	0.27
Afghanistan	208981 (134134-294141)	199.7 (187 to 212.8)	737 (479-1027)	0.5 (-3.4 to 4.4)	1.27	1.72	0.28
Djibouti	4119 (2626-5753)	78 (70.1 to 85.6)	473 (304-657)	-0.9 (-4.5 to 2.7)	0.82	1.8	0.42
Yemen	180267 (115752-254144)	175.9 (164.8 to 187.2)	749 (486-1044)	0.2 (-3.2 to 3.7)	1.29	1.71	0.45
Middle-income Countries							
Palestine	31910 (20345-44967)	159.2 (149.3 to 169.6)	760 (487-1061)	0.2 (-3.1 to 3.5)	1.31	1.72	0.45
Iraq	245414 (157277-342808)	135.5 (125.8 to 146)	754 (488-1052)	0.6 (-2.9 to 4.3)	1.29	1.73	0.47
Sudan	273586 (176305-381268)	124.5 (115.4 to 133)	760 (491-1059)	1.2 (-2.2 to 4.7)	1.28	1.72	0.52
Pakistan	1299007 (829027-1845495)	97.9 (91.4 to 105.3)	730 (468-1034)	-0.6 (-3.6 to 2.5)	1.23	1.62	0.52
Morocco	271235 (174130-382333)	64.1 (56.6 to 72.5)	770 (496-1084)	1.3 (-2.3 to 5.1)	1.25	1.71	0.62
Syria	130923 (84689-183571)	75.3 (67.9 to 84)	767 (496-1071)	1.4 (-2 to 5.3)	1.23	1.72	0.64
Egypt	674197 (433516-936625)	84.5 (77.6 to 92.4)	766 (494-1064)	0.9 (-2.5 to 4.9)	1.21	1.71	0.65
Jordan	55454 (35403-77551)	180 (166.9 to 193.4)	762 (492-1066)	1.9 (-1.7 to 5.5)	1.18	1.71	0.69
Tunisia	90158 (58559-124945)	64.4 (58 to 72.2)	755 (492-1048)	1.8 (-1.5 to 5.4)	1.17	1.73	0.70
Iran	653679 (425780-905798)	93.9 (85.8 to 102.5)	758 (497-1054)	2.3 (-1 to 5.7)	1.13	1.74	0.77
Libya	49500 (32133-69680)	75.6 (67.5 to 84.6)	771 (500-1085)	3.3 (-0.3 to 7.3)	1.13	1.71	0.81
Lebanon	47836 (30941-66597)	150.2 (140.8 to 160)	767 (496-1073)	0.5 (-2.9 to 3.9)	1.13	1.71	0.81
High-income Countries							
Oman	36619 (23376-50763)	234.9 (217.5 to 254.6)	700 (454-972)	-3.5 (-7.4 to 0.9)	1.07	1.72	0.72
Bahrain	11300 (7222-15841)	219.7 (205.8 to 234.5)	716 (463-1004)	-0.6 (-4.2 to 3.2)	1.08	1.7	0.74
United Arab Emirates	76336 (49496-107622)	513.9 (486.9 to 545.1)	659 (430-915)	-1.2 (-4.8 to 2.8)	0.98	1.73	0.77
Qatar	18937 (12171-26563)	445.1 (422.6 to 470)	678 (441-948)	-0.8 (-4.3 to 3.1)	1	1.71	0.79
Saudi Arabia	267107 (172575-373293)	146.1 (139.5 to 154.2)	794 (517-1110)	2.8 (0.8 to 4.9)	1.17	1.69	0.80
Kuwait	33859 (21841-48110)	126.6 (116.6 to 136.9)	745 (486-1050)	0.6 (-3.1 to 4.3)	1.09	1.71	0.83
EMR and global							
EMR	4702503 (3025930-6551569)	106.7^a^	746 (482-1039)	0.7^a^	1.21	1.69	N/A
Global	45121909 (29045835-62826904)	51.3 (49.7 to 52.8)	599 (386-833)	-0.2 (-0.8 to 0.4)	0.93	1.84	0.70

*YLD* years lived with disability, *EMR* eastern Mediterranean region

^a^ Uncertainty interval is not available

YLD numbers and rates of TTH in 2016, their changes from 1990 to 2016, and the ratio of observed to expected YLD rates on the basis of SDI in 2016 for EMR countries have been showed in Table 4. Iran, Oman, and Saudi Arabia had the highest increase in all-age YLD rate for TTH, while Somalia showed a slight decrease in all-age YLD rate for TTH. Similar to migraine, age-standardised YLD rates of TTH showed an overall consistency between 1990 and 2016 in EMR countries. Observed to expected age-standardised YLD rate ratio for TTH ranged from 0.85 in Djibouti to 1.42 in Iran. Djibouti and Somalia (both located in Eastern SSA and among low-income countries) were the only two countries with observed to expected age-standardised YLD rate lower than one. Locations with the highest level of age-standardised YLDs relative to the level expected on the basis of SDI were Iran, Palestine, Afghanistan, Iraq, Sudan, Yemen.

**Table 4:** YLD count, All-age YLD rates, and Age-standardised rates in 2016, and percentage change of YLD count, All-age YLD rates, and Age-standardised rates between 1990 and 2016 for TTH in EMR countries

Country	All-age counts		Age-standardised rates (per 100,000)				SDI
	2016	Percentage change between 1990 and 2016	2016	Percentage change between 1990 and 2016	Observed to expected ratio	F/M ratio	
Low-income Countries							
Somalia	7156 (4481-10831)	67.4 (58.5 to 77.6)	85 (54-128)	-1.3 (-6.1 to 4.2)	0.89	1.32	0.27
Afghanistan	35909 (22613-53732)	201.6 (186.6 to 218.1)	135 (85-200)	1.5 (-3.1 to 6.5)	1.4	1.39	0.28
Djibouti	706 (447-1058)	76.4 (64 to 90)	84 (53-125)	-3.2 (-8.5 to 3.1)	0.85	1.33	0.42
Yemen	31337 (19858-47513)	183.3 (165.5 to 205.2)	138 (88-207)	2.5 (-2.9 to 9.2)	1.39	1.4	0.45
Middle-income Countries							
Palestine	5548 (3486-8251)	164.6 (150.6 to 180.8)	139 (88-207)	1 (-3.6 to 6)	1.4	1.41	0.45
Iraq	42924 (27365-64414)	139.6 (126.8 to 154.5)	138 (88-206)	1.7 (-3.1 to 7.1)	1.39	1.42	0.47
Sudan	47952 (30469-71699)	129.9 (117.1 to 144.8)	140 (88-209)	3 (-2.1 to 9)	1.39	1.41	0.52
Pakistan	197112 (122210-292966)	98.4 (86.4 to 112.1)	114 (71-168)	-0.7 (-5.9 to 5.4)	1.13	1.28	0.52
Morocco	49517 (31448-73822)	73.3 (61.1 to 87.9)	142 (91-213)	3.3 (-2.3 to 9.4)	1.38	1.41	0.62
Syria	23274 (14638-34911)	82.9 (69.2 to 97.9)	142 (89-212)	3.5 (-2.3 to 9.9)	1.37	1.42	0.64
Egypt	121944 (77062-181851)	90.2 (79.4 to 102)	142 (91-211)	3 (-2.2 to 8.9)	1.36	1.42	0.65
Jordan	9934 (6276-14876)	191.3 (170.6 to 215.2)	142 (91-212)	3.7 (-1.6 to 9.6)	1.35	1.41	0.69
Tunisia	17148 (10721-25799)	73.1 (59.7 to 87.1)	144 (90-216)	3.3 (-2.3 to 9.4)	1.37	1.42	0.70
Iran	128660 (82720-188190)	104.2 (87.8 to 122.8)	152 (98-222)	4.1 (-1.8 to 11)	1.42	1.41	0.77
Libya	9053 (5661-13629)	84 (69.9 to 99.5)	144 (92-214)	4.4 (-0.9 to 10.8)	1.35	1.43	0.81
Lebanon	8862 (5586-13274)	160.8 (145.8 to 178.3)	144 (91-216)	2.8 (-2.3 to 8.7)	1.35	1.43	0.81
High-income Countries							
Oman	6862 (4261-10363)	259.7 (224.5 to 298.3)	135 (85-201)	0.9 (-6.7 to 9.2)	1.27	1.42	0.72
Bahrain	2112 (1327-3201)	234.2 (212.4 to 259.2)	136 (87-205)	1.4 (-4.1 to 6.8)	1.29	1.42	0.74
United Arab Emirates	15103 (9215-23288)	542.9 (493.5 to 600.3)	132 (82-198)	0.5 (-5.2 to 6.8)	1.24	1.42	0.77
Qatar	3540 (2191-5423)	464 (423.6 to 505.7)	130 (82-194)	2.1 (-3.5 to 8.2)	1.22	1.45	0.79
Saudi Arabia	44858 (28544-65886)	160.2 (140.2 to 180)	137 (88-199)	5.3 (-0.3 to 10.5)	1.28	1.39	0.80
Kuwait	6281 (3929-9644)	138.3 (121.4 to 157.8)	141 (89-213)	2.6 (-2.9 to 8.9)	1.32	1.42	0.83
EMR and global							
EMR	815789 (516411-1221998)	112.8^a^	134 (85-198)	2.5^a^	1.29	1.38	N/A
Global	7195122 (4614628-10499903)	53.2 (47.5 to 58.4)	96 (62-140)	-0.2 (-2.5 to 1.9)	0.91	1.48	0.70

*YLD* years lived with disability, *TTH* tension-type headache, *EMR* eastern Mediterranean region

^a^ Uncertainty interval is not available

## Discussion

This is the first study to investigate the prevalence and burden of headache in EMR. Our study provides a comprehensive assessment of the values and trends of prevalence and burden of primary headache disorders in EMR countries and their trends from 1990 to 2016. Our findings are of great value to health professionals and their efforts to reduce the burden of headache in the region. Indeed, our results could be used for planning and implementing programs to address this burden.

From 1990 to 2016, despite the overall consistent age-standardised YLD rates of headache, the all-age YLD rates and the total number of people suffering from headache has substantially increased. This finding is consistent with the global growing burden of non-communicable diseases [4]. Consistent age-standardised YLD rates along with the increase of number of people affected by headache indicates the role of population growth and ageing as the main factors of the increase in the burden of headache disorders.

Burden of headache was generally higher in the EMR countries compared to global average. Risk factors for progression of episodic migraine to chronic migraine can explain a part of the higher burden of headache in the EMR. Studies suggest that primary chronic migraine is rare, and chronic migraine usually progresses from episodic migraine. Gradual increase in frequency of episodic migraine attacks progresses to chronic migraine with an annual rate of 2.5% [29, 30]. The most important risk factors for progression of episodic migraine to chronic migraine are medication overuse for migraine attacks, obesity, depression, anxiety, and stressful life events [31, 32]. The higher rates of depression and anxiety in the EMR compared to the rest of the world can play a role in the higher burden of migraine in this region. In 2013, mental disorders contributed to 19.0% of non-fatal burden in the EMR, in comparison to 17.4% of global non-fatal burden [33]. Stressful life events can be another contributor of the higher burden of migraine in the EMR. For example, countries with ongoing or recent conflicts such as Palestine, Iraq, Yemen, Sudan, and Afghanistan had the highest observed than expected age-standardised YLD rates of migraine on the basis of their SDI. However, given the limited data sources from the EMR countries, the role of risk factors of chronic migraine in higher burden of headache in the EMR should be interpreted cautiously.

The EMR had greater than expected age-standardised YLD rates on the basis of SDI for headache, and the gap between observed and expected rates remained generally unchanged from 1990 to 2016. In contrast, worldwide observed age-standardised YLD rates were lower than the expected values during the same period. Constant high ratio of age-standardised YLD rates relative to that expected in the EMR suggests poor performance of health service in terms of headache outcomes relative to the development status, reflecting a need for additional attention to the reasons of this underperformance and policy implications for health reforms in EMR countries.

YLD rate of migraine and TTH were highest between ages 25 and 54 years, which is consistent with the literature [34]. However, the burden of headache is still considerable in 5–24 and 55–74 year old age groups.

Estimated all-age prevalence rates for migraine and TTH in the EMR were higher compared with the global rates. A review on epidemiological studies between 1988 and 2005 estimated that the global prevalence of migraine and TTH were 10% and 38% respectively [1]. However, studies have shown a wide variation in the prevalence of primary headache disorders, partly because of the differences in methodology. Particularly for TTH, case definition has been a challenge; for example, variation in frequency and duration of TTH and its overlap with migraine may have led to differences in prevalence [35]. Disparity in prevalence rates, reported by studies with different methodology, made them non-comparable over time and across geographic areas. GBD, however, is a systematic approach that has the important advantage of providing comparable prevalence and burden over time and by geography.

Our findings have important health service implications. The significant and increasing non-fatal burden of headache inform policy makers and health care providers of EMR countries that primary headache should be a health care priority, and intervention strategies focusing on improvement of diagnosis and treatment of headache must be implemented. Primary headache is one of the leading causes of disability; meanwhile, effective and low-cost treatments are available for most patients with primary headache (including patients with migraine and TTH). In most cases, patients with primary headaches can be diagnosed and treated by the primary health-care physicians. Moreover, high prevalence of primary headaches across a wide range of ages necessitates interventional strategies that, in the first place, target the primary health service. Better education of health-care professionals, especially those who work in primary health-care services, is the most essential part of interventional strategies to reduce the burden of headache. Health-care professionals should be educated to properly diagnose and treat patients with primary headache disorders. Providing diagnostic and treatment guidelines, especially for non-expert healthcare providers is also a practical method especially for the low-income countries [11].

The implication of these findings is that investment in interventional strategies for diagnosis and treatment of headache will lead to a considerable reduction in the healthcare costs and the burden of headache. Our study calls for more population-based surveys investigating the prevalence and severity of primary headache disorders in EMR countries. Population-based and disease-specific surveys providing better data coverage on headache epidemiology in EMR are needed for more accurate estimates of primary headache burden in the region.

Quality of life and financial impacts are important contributors of the burden. For example, with a mean per-person annual cost of €1222, the annual total cost of migraine has been estimated to be €111 billion in Europe in 2009 [36]. Another study estimated the mean per-person annual cost of episodic and chronic migraine at $2649 and $8243 in the US in 2013 [37]. Furthermore, studies from Sweden [38], then US [39], and the UK [40] have reported that migraine has a considerable negative effect on family quality of life. In this study, although we estimated the most important contributors of the headache’s burden, we did not capture its costs and quality of life.

This study has some limitations. The main concern is the lack of adequate data and poor quality of the available data from the EMR. Since accessible data sources for prevalence of migraine and TTH in EMR are scarce, we were able to use data from only few of the EMR countries. In comparison to GBD 2013, with prevalence data from only one EMR country (Iran), GBD 2016 is an obvious improvement; however, there is still no reliable data from most of the EMR countries. Unfortunately, the region is facing several health challenges which divert attention from headaches in the mind of many health professionals. However, we hope that our study will increase the attention to this large burden and lead to improved data. Second, although we estimated prevalence and YLDs of the primary headache disorders with considerable burden (including migraine, TTH, and MOH - as a sequel of the first two syndromes), we could not include all primary headache disorders classified in ICD-10 classification [41]. Cluster headache, for example, was the most important headache syndrome that we did not capture in this GBD iteration.

## Conclusion

Findings from this study show that primary headache disorders are a large cause of disability in the EMR. Despite generally unchanged age-standardised YLDs, YLD numbers increased substantially between 1990 and 2016 in the EMR-mostly due to the population growth and ageing. Our findings inform policy makers of the EMR countries that headache is a health care priority, and preventive and management interventions must be implemented to address the growing burden of headache in this region. More studies are needed to provide more accurate data on the prevalence and severity of primary headache disorders in EMR as well as more efficient preventive and management methods to reduce the burden of headache.

## Additional files

Additional file 1: (XLSX 26 kb)

Additional file 2: (XLSX 23 kb)

## Abbreviations

DALY: Disability-adjusted life years
DW: Disability weight
EMR: Eastern Mediterranean Region
GBD: Global Burden of Disease Study
IHME: Institute for Health Metrics and Evaluation
LDI: Lag-distributed income
MOH: Medication overuse headache
SDI: Socio-demographic Index
SSA: Sub-Saharan Africa
TFR: Total fertility rate
TTH: Tension-type headache
WHO: World Health Organization
YLD: Years lived with disability
YLL: Years of life lost
